# You are what you eat and how you digest it! A discussion on inflammatory efferocytosis

**DOI:** 10.3389/fcell.2023.1132696

**Published:** 2023-02-09

**Authors:** Maria C. Tanzer

**Affiliations:** Department of Proteomics and Signal Transduction, Max Planck Institute of Biochemistry, Martinsried, Germany

**Keywords:** efferocytosis, inflammation, LC3-associated phagocytosis, cell death, phagolysosome

## Abstract

Efferocytosis is a process by which phagocytes remove dead or dying cells. It is considered anti-inflammatory, as the removal process reduces potential inflammatory molecules originating from dead cells and results in the reprogramming of macrophages to an anti-inflammatory state. However, engulfment of infected dead cells, deregulated phagocytosis and perturbed digestion of apoptotic bodies induce inflammatory signalling pathways during efferocytosis. The affected inflammatory signalling molecules and the mechanism of activation are largely unknown. I discuss how the choice of dead cell cargo, the type of ingestion, and the digestion efficiency can influence phagocyte programming in the context of disease. I also present the latest findings, highlight knowledge gaps, and propose selected experimental approaches to fill them.

## Introduction

The adage ‘Nothing is certain but death’ (modified from Benjamin Franklin) is valid for all cells in our body. From their formation to their last signalling events, cells balance pro-survival and pro-death signalling triggered by external and internal cues. Pathogens, DNA damage, and a variety of other stressors can sway this balance (Galluzzi et al., 2018). While infected or damaged cells can be detrimental to the body, excessive cell death can also be problematic, especially in the inflammatory context (Rothlin et al., 2021). The number of dead cells, location, and cell death type substantially influence the inflammatory potential. The tumour necrosis factor (TNF), a pro-inflammatory cytokine, can induce apoptosis–considered an immunological silent cell death type–and necroptosis, an inflammatory cell death type (Murphy et al., 2013; Tanzer et al., 2021; Tanzer, 2022). Infection by many pathogens can also induce a highly inflammatory form of cell death, termed pyroptosis (Bergsbaken et al., 2009). Endogenous inhibitors of cell death, such as cellular FADD-like Il-1β-converting enzyme inhibitory protein (cFLIP), inhibitors of apoptosis (IAPs), myeloid leukemia cell differentiation protein (MCL-1) and B-cell lymphoma 2 (BCL-2) are effective (Vaux et al., 1988; Chang et al., 2002; Silke and Meier, 2013). However, overpowering cell death signals lead to excess cell death. Therefore, the efficient removal of dead cells is essential to prevent pathologic inflammation and sustain homeostasis of our bodies.

**FIGURE 1:**
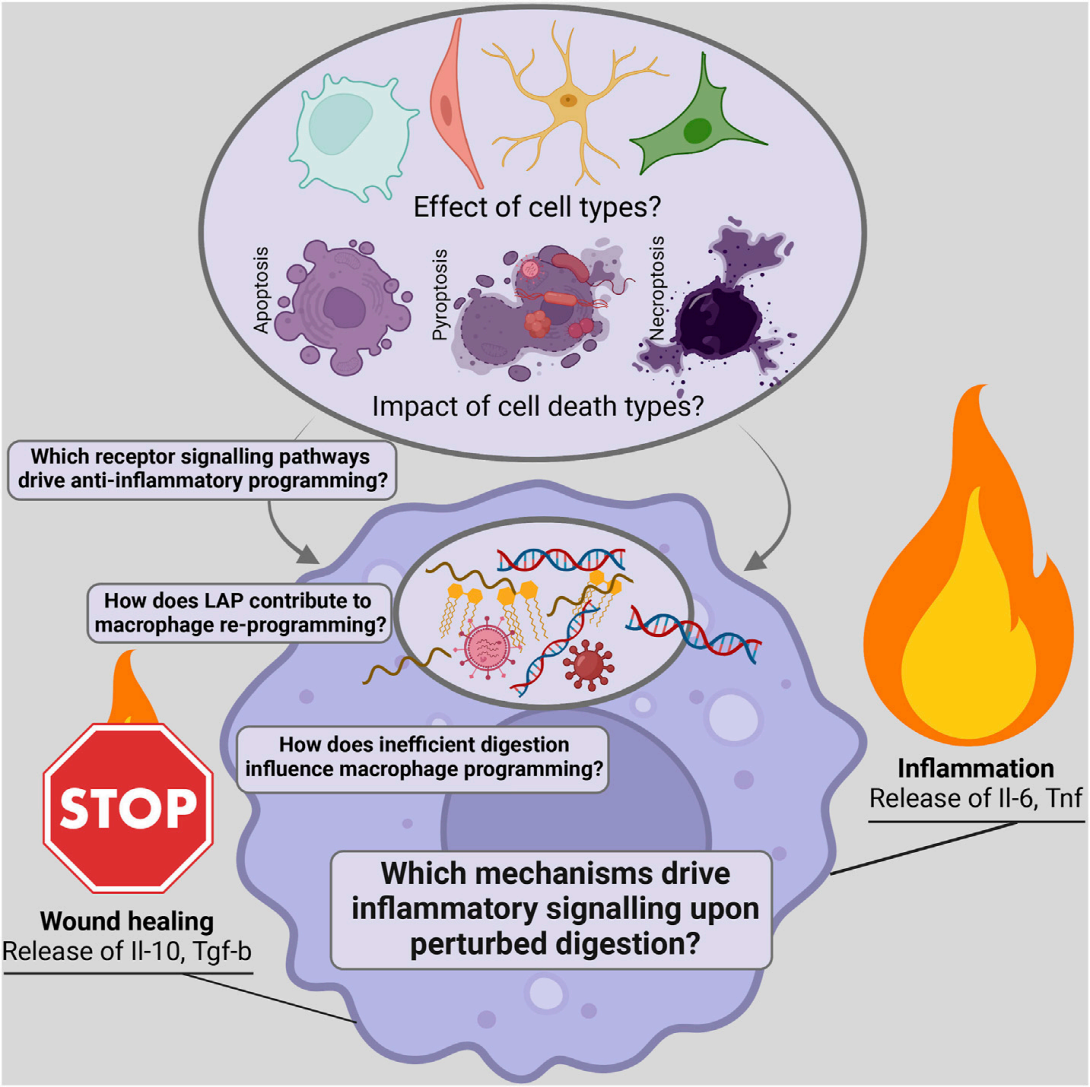
Scheme depicting open questions on the influence of dead cargo and the identification of signalling pathways regulating the inflammatory potential of efferocytosis.

The removal of dead cells is called efferocytosis and is primarily mediated by macrophages. The efficient efferocytosis of dead cells is essential for wound healing and for containing inflammation. Macrophages undergo a programming switch towards an anti-inflammatory state with the release of interleukin 10 (IL-10) and transforming growth factor beta (TGF-β) (Voll et al., 1997). Upon sensing phosphatidylserine (PS) exposure on dead cells, macrophages initiate engulfment *via* LC3-associated phagocytosis and induce anti-inflammatory programming (Fadok et al., 1992; Korns et al., 2011). Many upstream events, such as ‘find-me’ signals released by the dead cell to attract macrophages, have been identified. PS receptors on macrophages such as Tyro 3, Axl and Mer (TAM), transmembrane immunoglobulin and mucin domain 1 and 4 (TIM-1 and 4), CD300a,b,f, stabilin-1 and 2, the receptor for advanced glycosylation end products (RAGE), brain-specific angiogenesis inhibitor 1 (BAI 1) are partially redundant, but overall essential for engulfment (Elliott et al., 2017). Little is known about the relationship between cell type, cell death, and efferocytosis efficiency, as well as the release of anti-inflammatory factors (Figure 1). Additionally, the regulation of engulfment and digestion and the role these processes play in reprogramming macrophages is still poorly understood. Even less is known about the mechanisms activated when efferocytosis is disrupted and pro-inflammatory signalling is triggered. The perturbation of engulfment by Rubicon deficiency, an essential member of the LC3-associated phagocytosis pathway, and digestion seen in patients with mutated deoxyribonuclease II (DNase II), generate pro-inflammatory signalling (Okabe et al., 2005; Cunha et al., 2018). To better understand these signalling pathways, further research must be conducted to uncover the events that inhibit anti-inflammatory and drive pro-inflammatory signalling during efferocytosis, which could offer therapeutic targets for treating inflammatory and infectious diseases (Figure 1).

### The menu–dead cells and their inflammatory potential

Apoptosis, necroptosis and pyroptosis are the main cell death types involved in inflammation, activated by cytokines and pathogens. Necroptosis is mediated by MLKL activation, and pyroptosis is mediated *via* gasdermin D activation. Both are lytic cell death pathways. (Sun et al., 2012; Murphy et al., 2013; Kayagaki et al., 2015; Atkin-Smith, 2021). In contrast, apoptotic cells stay intact and active caspases degrade inflammatory molecules before their leakage into the extracellular space. Numerous reports investigate the inflammatory potential of these different cell death types by studying inflammatory factors released by dying cells (Dinarello, 1997; Orozco et al., 2019; Tanzer et al., 2020; Phulphagar et al., 2021). Pyroptosis, for example, leads to the release of many inflammatory mediators, including the highly pro-inflammatory cytokines cleaved IL-1B and IL-18 (Dinarello, 1997; Phulphagar et al., 2021). The inflammatory potential of TNF-induced apoptosis and necroptosis is less clear. Apoptotic T-cells release anti-inflammatory factors including TGF-β, IL-10 and ATP (Szondy et al., 2017). Nevertheless, in a global analysis of proteins released by these cell death types *in vitro*, we see prolonged production of cytokines by apoptotic cells. In contrast, during the early stages of necroptosis, dying cells release various cellular components such as lysosomal proteins (Tanzer et al., 2020).

Cell death types not only differ in their inflammatory potential based on the release of different inflammatory factors, but also differentially impact the removal of dead cells. A macrophage’s perturbed or deficient ability to detect and remove dead cell populations increases the dead cells’ inflammatory potential. Nevertheless, less focus has been placed on how different cell death pathways influence removal efficiency. The cell death type affects the recruitment of macrophages through the release of find-me signals, including metabolites, chemokines and growth factors (Ravichandran, 2010). Systematic comparisons of proteomic and metabolomic analyses of dead cell supernatants and *in vivo* analyses will give more insight into the recruitment ability of cells dying *via* different cell death pathways.

A classical hallmark of apoptosis is the exposure of PS, which is also the main trigger for engulfment. Upon apoptosis, certain flippases such as ATPase phospholipid transporting 11c (ATP11c), are inhibited and scramblases such as XK-related protein 8 (XKR8) are activated by active caspases (Nagata et al., 2016). Interestingly, necroptotic and pyroptotic cells also demonstrated increased PS staining, but due to membrane permeabilisation, this might not be specific to the outer leaflet (Zargarian et al., 2017; Atkin-Smith, 2021). Efferocytosis efficiency of necroptotic and pyroptotic cells was reduced. This could be due to the increased expression of engulfment inhibitors like cluster of differentiation 47 (CD47) and the lack of other pro-engulfment factors on necroptotic cells (Gardai et al., 2005). Another factor for the optimal stimulation of engulfment receptors could be the distribution of PS on the outer leaflet (Kern et al., 2021). Considering the significant differences in cell morphology between apoptosis, necroptosis and pyroptosis, differences in PS distribution are very likely.

Besides engulfment and recruitment, digestion efficiency will vary between different cell corpses. Digestion of smaller apoptotic bodies will likely be faster than that of large necroptotic cells (Atkin-Smith and Poon, 2017). The variable disassembly of cells could also influence their digestion. In contrast to necroptotic cells, apoptotic cells activate caspases that cleave many intracellular inflammatory components. These differences affect the reprogramming of macrophages. Consequently, macrophages engulfing necroptotic cells release increased pro-inflammatory cytokines (Zargarian et al., 2017). Comparing signalling activation between the engulfment of apoptotic *versus* necroptotic or pyroptotic cells could elucidate pathways important for anti-inflammatory reprogramming.

The recruitment, engulfment, digestion and inflammatory programming will also be affected by the cell type of the dying cell and the tissue-specific macrophages, such as Kupffer cells in the liver and microglia in the brain. Investigations of dead cells and macrophages within these tissues, for example, by combining laser microdissection with single-cell proteomics, will be necessary to regulate efferocytosis effectively in tissue-specific diseases (Mund et al., 2022).

### When ingestion is deregulated–LC3-associated phagocytosis

Activating LC3-associated phagocytosis (LAP) is essential for anti-inflammatory reprogramming during efferocytosis (Heckmann and Green, 2019). In contrast to classical autophagy, the engulfment of corpses leads to the recruitment of LAP-specific proteins such as Rubicon and nicotinamide adenine dinucleotide phosphate (NADPH) oxidase 2 (NOX2) to the single-membrane phagosome (Martinez et al., 2015). Recruitment of vacuolar protein sorting 34 (VPS34) to the complex induces the production of phosphatidylinositol 3-phosphate (PI3P), which is essential for LC3 binding to the phagosomal membrane (Heckmann and Green, 2019). LC3 association facilitates phagosome fusion with the lysosome and Rab-GTPases enhance phagosome maturation (Fazeli and Wehman, 2017). Consequently, the digestion efficiency of corpses and certain phagocytosed pathogens is increased, which is believed to reduce inflammation, contributing to the induction of anti-inflammatory mediators (Heckmann and Green, 2019). Cunha et al. demonstrated that perturbation of LAP by knocking out LAP-specific components, including Rubicon, induced type I interferon responses *via* the activation of Sting1 through unknown mechanisms (Cunha et al., 2018). This inflammatory response reduced tumour growth of a melanoma cell line *via* activation of T-cells. The LAP machinery could therefore serve as a target for anti-tumour therapy. Another study reported Rubicon as an inhibitor of the antiviral response by directly interacting with interferon regulatory factor 3 (Irf3) and inhibiting its dimerisation (Kim et al., 2017). Pena Martinez et al. speculated on Rubicon’s role in localising and inhibiting the NLR family pyrin domain containing 3 (NLRP3) inflammasome (Pena-Martinez et al., 2022). Another report demonstrated Rubicon interacting with p22phox, an NADPH oxidase subunit. This interaction increased reactive oxygen species (ROS) production, cytokine expression and pathogen clearance (Yang et al., 2012). In the future, it would be interesting to dissect Rubicon’s role in a LAP-dependent and independent context. Two Saudi Arabian families have been identified as harbouring Rubicon mutations, which obstruct its endolysosomal localisation, resulting in ataxia as the primary symptom in afflicted family members (Assoum et al., 2010; Seidahmed et al., 2020). More investigation into immunological abnormalities upon infection or in the context of cancer could further elucidate its function in a physiological setting.

Since the first description of LAP in 2007, many questions about LAP regulation and its physiological impact remain (Sanjuan et al., 2007). Rubicon complex recruitment to the phagosome depends on the engagement of PS receptors such as TIM4, but the exact mechanisms are unknown (Martinez et al., 2011). Regulations of LC3 binding to the phagosome downstream have also to be determined. Intact LAP is a prerequisite for efficient cargo digestion and subsequent inhibition of inflammation. However, it is unclear how LAP signalling regulates phagolysosome acidification, major histocompatibility complex (MHC) class II presentation and digestion of diverse cargo components (Romao and Munz, 2014). Future investigations that analyse ingested material and compare the signalling activation of LAP-deficient cells to that of wild-type cells could shed light on the role of LAP in macrophage reprogramming during efferocytosis.

### Digestion, digestion, digestion: The leaky phagolysosome in health and disease

The last step of efferocytosis involves the digestion of the cellular corpse. The majority of dead cell digestion occurs in the phagolysosome, where pH levels are low, and active proteases, DNases and other digestive enzymes are present (Howell et al., 2003; Yang and Wang, 2021). Efficient digestion is a prerequisite for efficient efferocytosis and is essential to prevent inflammation. Two decades ago, the Nagata Lab showed that DNaseII-deficient mice died due to defective erythropoiesis induced by perturbed digestion of old erythrocytes (Kawane et al., 2001). Later, they demonstrated that transfer of DNaseII-deficient bone marrow cells induced arthritis and that macrophages were responsible for the phenotype (Kawane et al., 2010). Besides deficient digestion, deficient engulfment was also linked to disease. Increased auto-antibodies were detected in TAM-deficient mice. Lupus-like autoimmune disease features were observed upon lack of the scramblase Xkr8, which regulates PS exposure of apoptotic cells or in milk fat globule epidermal growth factor 8 (Mfg-e8)-deficient mice lacking Mfg-e8, a PS-binding protein mediating integrin binding and engulfment (Lu and Lemke, 2001; Hanayama et al., 2004; Kawano and Nagata, 2018).

The origin of inflammatory signalling upon hampered efferocytosis is challenging to define but essential for developing effective anti-inflammatory treatment. Reduced removal of dead cells leads to accumulating inflammatory and immunogenic intracellular material such as DNA in the extracellular space. Extracellular DNA is a prime example of a significant inflammatory mediator. DNA clearance is reduced during sepsis and is linked to COVID-19 severity (Aramburu et al., 2022). DNA in the extracellular space activates the B-cell response leading to increased anti-DNA antibodies in the plasma. Extracellular DNases are, therefore, essential to reduce inflammation and are heavily studied in the context of lupus (Martinez Valle et al., 2008).

Perturbed ingestion and digestion of dead cells not only lead to the release of inflammatory material in the extracellular space, but also activate inflammatory pathways within the efferocytosing cell. Several studies indicated that phagolysosomes are not entirely sealed and leakage of undigested material could trigger inflammation (Okabe et al., 2005; Cunha et al., 2018). Although it has not been experimentally demonstrated thus far, undigested DNA is believed to leak into the cytosol in DNaseII-deficient macrophages and be responsible for the secretion of inflammatory cytokines (Okabe et al., 2005). Consistently, Cunha et al. detected Sting1 activation and interferon production in mice with LAP-deficient macrophages (Cunha et al., 2018). Earlier, it was shown that transfection of DNA from apoptotic and necrotic cells induced Sting1 activation in macrophages (Ahn et al., 2012). The mechanism of DNA release from the phagolysosome is unknown. Oxidative stress has been found to induce lysosomal rupture (Terman et al., 2006). Whether this contributes to phagolysosome leakage of efferocytosing macrophages has yet to be determined. Engulfment of large-sized cell corpses could put mechanical strain on the phagolysosomes and contribute to leakage. These explanations entail phagolysosomal leakiness in efferocytosing wild-type macrophages, which do not exert pro-inflammatory signatures. How wild-type cells circumvent the pro-inflammatory response would be interesting to address. During intact efferocytosis in wild-type cells, digestion will be efficient, and leakage of undigested material will be minimal but present. Upon leakage of undigested material, mechanisms inhibiting Sting1 activation and other inflammatory pathways must be in place. How perturbed engulfment and digestion override these anti-inflammatory programs is unknown.

It is also unclear whether other molecules of dying cells, such as proteins, lipids and other metabolites leak into the cytosol and whether they are also triggering an inflammatory response. Zhang et al. demonstrated that fatty acids from engulfed apoptotic cells activated mitochondrial respiration to ensure NAD^+^ production, Sirtuin activation and IL-10 upregulation upon efferocytosis (Zhang et al., 2019). This implies that fatty acids exit the phagolysosome. Considering these discoveries, a particular focus should be set on the impact of different cargo on macrophage programming. Cells derived from different tissue and undergoing different cell death types will differentially activate macrophage receptors and influence macrophage programming based on cytosolic exposure of cellular debris.

Pathogens within dead cells can also access the cytosol. Certain pathogens, such as *Staphylococcus aureus* and *Salmonella typhimurium,* are directly phagocytosed by macrophages, undergo LAP and invade the cytosol through the phagolysosome (Herb et al., 2022; Mehrotra and Ravichandran, 2022). Other pathogens could not infect macrophages if it was not through another infected dead cell that serves as a Trojan horse. Efferocytosis of apoptotic cells infected by SARS-CoV-2 induced upregulation of pro-inflammatory cytokines (Salina et al., 2022). Viral RNA could thereby enter the cytosol and induce an antiviral response. While not every virus investigated induced the production of pro-inflammatory cytokines, efferocytosis of apoptotic bodies infected with the influenza A virus led to increased viral production (Atkin-Smith et al., 2020; Salina et al., 2022). Systematic examinations of signalling activated upon engulfment of dead cells infected with selected pathogens could help determine the contribution of phagolysosome leakage in various infectious diseases. In the context of viral infections, the macrophage could activate antiviral signalling and act as a new host cell by producing viral proteins. Intracellular localisation studies using proteomics and metabolomics approaches to identify peptides and metabolites of pathogens within macrophages in combination with high resolution microscopy will elucidate the understudied aspects of pathogen-macrophage interactions.

## Concluding remarks

Even though efferocytosis exemplifies anti-inflammatory processes, like most immune processes, it is susceptible to perturbations. Due to its complexity, efferocytosis can be deregulated at many levels. To gain a better understanding of the precise signals that lead to the anti-inflammatory programming of macrophages, more research is necessary. The roles of various receptors in reprogramming macrophages upon efferocytosis has not been dissected precisely. To achieve this, tissue specific receptor deletion is required, as macrophage receptor expression is variable dependent on macrophage location. Additionally, macrophages that are resident in different tissues are exposed to different cell types undergoing different cell death processes. This will influence the engulfment and digestion efficiency and will pose variable metabolic challenges on macrophages. The effect of efferocytosis of dead cells with variable metabolic compositions on macrophage reprogramming has to be investigated, especially in context of disease. Also, pathogens within dead cells may be able to manipulate efferocytosis and the inflammatory status of macrophages for their own benefit. State-of-the-art technology, such as spatially resolved high-sensitive metabolomics and proteomics including the analysis of post-translational modifications, will uncover regulators of efferocytosis *in vitro*, in mouse models, and patient samples.

## Open access statement

Open access for this article was enabled by the participation of Max Planck Digital Library.
